# Impact of internet plus health education on urinary stoma caregivers in coping with care burden and stress in the era of COVID-19

**DOI:** 10.3389/fpsyg.2022.982634

**Published:** 2022-11-30

**Authors:** XuFei Fan, HaiYan Li, LiYa Lai, XiaoFeng Zhou, XiangXiang Ye, HaiNiao Xiao

**Affiliations:** ^1^Department of Hernia and Abdominal Surgery, The First Affiliated Hospital of Wenzhou Medical University, Wenzhou, China; ^2^Department of Urology, The First Affiliated Hospital of Wenzhou Medical University, Wenzhou, China

**Keywords:** internet, health education, urinary ostomy, caregiver, pressure coping

## Abstract

**Objective:**

To explore the impact of “Internet Plus Health Education” on coping with care burden and pressure in urinary stoma caregivers in the era of COVID-19.

**Materials and methods:**

Eighty caregivers of patients with urinary ostomy were equally randomized to experimental and control groups. Caregivers in the experimental group received digital nursing education intervention, which involved nursing intervention of Internet Plus Health Education (IPHE), and those in the control group received conventional care instructions. Six months later, care burden and emotional pressure were assessed in all caregivers using the Zarit Caregiver Burden Interview (ZBI) and the Simplified Coping Style Questionnaire (SCSQ).

**Results:**

Before the intervention, the ZBI and SCSQ scores were comparable between both groups (*p* > 0.05). After the intervention, the ZBI scores in the experimental group were significantly higher than in the control group and vice versa for SCSQ scores (*p* < 0.01). Furthermore, after the intervention, the family care satisfaction scale (FCSS) of the experimental group was significantly higher than the control group.

**Conclusion:**

Providing “Internet Plus Health Education” to urinary stoma caregivers can reduce their care burden and enhance their pressure-coping ability in the COVID-19 era.

## Introduction

Bladder urothelial carcinoma (BUC) accounts for about 5% of all cancer-related deaths worldwide (Brincks et al., 2013) and is the most common malignant tumor of the urinary system in China (Xu et al., 2022). Radical bladder resection with urinary diversion remains the mainstay of treatment of malignant bladder tumors (Jeong et al., 2022), but the patient needs to wear ostomy bags postoperatively (Li et al., 2014). Given that the secretions in the ostomy bag consist mainly of fluids, more complex and meticulous nursing care is required compared to solid secretions in patients that underwent colostomy (Chen et al., 2021).

Since COVID-19 was first reported in December 2019 (Lee et al., 2014), it has become extremely challenging to care for ostomy bags after the urostomy patient is discharged from the hospital (Jiang et al., 2021). Professional nurses usually implement urinary stoma care during hospitalization, but after discharge, most of the professional nursing work is performed by the patient’s caregiver (Rothstein, 1986; Zganjar et al., 2021). Indeed, the caregiver must cope with the psychic fear and rejection upon first contact with an ostomy and face confusion due to a lack of knowledge and skills about stoma care. For these reasons, they often feel self-accused (Rothstein, 1986). In the long run, it increases the caregiver’s physical, psychological and social burdens (Zganjar et al., 2021), affecting his/her attendance on the patient and his/her own psychosomatic health. Accordingly, it is necessary to improve the caregiver’s coping capability.

The internet has become an important medium for patients and their families to improve their healthcare knowledge (Mieronkoski et al., 2017; Norton et al., 2022). In this respect, the internet can provide the stoma caregiver with a multi-dimensional, simple, quick and effective approach to health education, embodying a new way of thinking in the development of modern nursing care (Chute et al., 2022). This study proposes a method of using digital nursing education to improve the nursing ability of caregivers of urostomy patients, mainly through WeChat (a social media application), online video, cloud storage, and other channels to establish contact and education with caregivers, an approach termed Internet Plus Health Education (IPHE).

**FIGURE 1:**
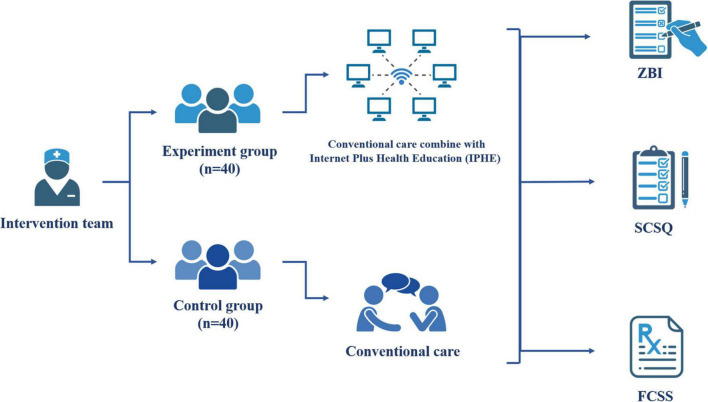
The experimental flow chart of this study. Made using BioRender.

Existing research has mostly focused on the self-education of urostomy patients, pre-ostomy education for bladder cancer patients (Wulff-Burchfield et al., 2021), analysis of postoperative quality of life for patients with ostomy (Mo et al., 2022), and effective evaluation of Ostomy Self-Management Telehealth (OSMT) (Grant et al., 2022), etc. Herein, we selected caregivers of patients with urostomy as study subjects and explored the effectiveness of using the internet to provide urinary stoma caregivers with health education to reduce their care burden and increase their coping ability in the context of COVID-19 (Figure 1).

## Manuscript formatting

### Inclusion criteria

According to the inclusion and exclusion criteria (Chute et al., 2022), 96 urinary stoma caregivers of patients who underwent urinary ostomy for urinary malignant tumors in the department of urology of a tertiary hospital in Wenzhou, China between January 2020 and December 2020. Among them, 80 participants met the inclusion criteria and obtained informed consent, and there were no participants lost to follow-up during the 6-month study period. The inclusion criteria included (1) caregivers of patients who underwent urinary ostomy for malignant bladder tumors with primary school and higher educational backgrounds and could skillfully use WeChat, QQ and other IRC, as well as mobile application software; (2) family members of the patients, including their spouses, sons, daughters and siblings; (3) caregivers aged from 18 to 70 years who provided care for more than 2 weeks, totaling > 40 h in a week including the time of companionship, who were expected to do the care job for more than 3 months; and (4) caregivers who volunteered to participate in the study. The exclusion criteria were (1) caregivers with mental or cognitive disorders; (2) caregivers with no kinship with the patients; and (3) caregivers with severe illnesses. The 80 caregivers included were divided into experimental and control groups according to the random number table method.

The sample size of this study was determined by the expected exposure odds ratio OR; the control group had an exposure rate p0. The probability of making a Type I error was α, and the expected test power was (1-β), where β refers to the probability of a Type II error. In this study, we used a significance level (α) of 0.05 and β = 0.10 (for 90% power).

## Materials and methods

### Intervention in the control group

The caregivers in the control group received conventional care instructions (Fuschi et al., 2021). Briefly, they were provided knowledge on stoma care and postoperative care of patients who underwent surgery for malignant bladder and were instructed how to change the stoma bag. In addition, they were provided an ostomy knowledge education manual and notified of the follow-up schedule of the patients.

### Intervention in the experiment group

In addition to the conventional care instructions provided to the control group, caregivers in the experimental group also received Internet Plus Health Education (IPHE) as follows:

An intervention team consisted of a urologic surgeon with a Ph.D. Degree as the team leader, an enterostomal therapist (ET), a registered nurse (RN), 3 nurse practitioners (NPs), and one of the researchers of the present study. According to the “Urinary Stoma Care and Rehabilitation Guidelines”, the caregivers in the experimental group received standardized training and performance evaluation, and only those who passed the evaluation could participate in the study.

After signing the informed consent form, the eligible caregivers were allowed to participate in this study. A complete file of the caregivers was set up. First, the caregivers were informed of the study’s objective and details of the intervention during hospitalization to increase their compliance and sense of cooperation. A WeChat group was established between the caregivers and the medical care workers (MCWs), and teaching materials were shared *via* E-mail and Baidu cloud storage. Each researcher ensured that every eligible caregiver recruited in this study could use the internet tools.

Care evaluation during hospitalization: Care evaluation during hospitalization was conducted through a questionnaire-based investigation, including knowledge of urinary stoma care, the current status of the caregivers in coping with care burden and pressure and the health demands of the patients and caregivers. In addition, the caregivers were gathered to watch an instructional video on stoma care.

Care evaluation 1 month after discharge: Instructions about changing the ostomy bag were provided *via* the IPHE mode. When the caregivers returned home and were confronted with the lack of knowledge about urinary stoma care during this period, videos about stoma change were provided *via* the Baidu cloud storage, while the nurses of the hospital gave related instructions *via* voice communication on the WeChat platform. The video demonstrated stoma manipulation, which involved replacing the stoma chassis and bag and emergency management in case the stoma chassis fell off. The researcher assisted the caregivers in solving problems they encountered.

Two months after discharge: The main task in this period was to dredge psychological problems and resolve ostomy care difficulties of the caregivers *via* the IPHE mode. Within 2 months, most caregivers had learned how to do the change procedures independently, but some did not know how to cope with the patients’ negative emotions. In this situation, the MCWs would contact the caregivers *via* WeChat to know their concerns and psychological problems to help the caregivers dredge the patients’ negative emotions. For instance, the MCWs would advise the caregivers to think empathically when they raised questions like “what can I do when the patient doesn’t like going out?”, “What should I do when the patient always loses his temper?” In this way, they could understand the patient’s physical and psychological behaviors and enhance communication, which would also attenuate their care burden and pressure.

Three months after discharge: Feedback evaluation and cognitive reconstruction were the main tasks of IPHE during this period. At 3 months, a questionnaire-based investigation was implemented *via* WeChat to collect caregivers’ data to learn about their cognitive deviations in stoma care, their level of knowledge and skills in stoma care, and the presence or absence of their negative emotions during the process of stoma care for the sake of raising their capability of emotional control and helping them with psychological construction.

Four months after discharge: The main task of IPHE during this period was to intensify technical training in stoma care. Based on the previous evaluation results, the MCWs would help the caregivers to improve their care skills and help the patients adapt to the bag-wearing life. During the intervention process, the MCWs would answer questions about complication management, such as “What should I do when a red circle appears over the skin around the ostomy?”, “Why, the stoma is recessed.” These questions were answered *via* WeChat, and the caregivers were told how to solve these problems or advised to seek medical help.

Five months after discharge: The main task in this period was to build a WeChat platform to share the experience in stoma care. Using the WeChat group, the MCWs would encourage the caregivers to share their experiences in stoma care and recommend stoma care knowledge networks and videos of the caregivers in improving the quality of home care and the quality of life of the patients.

Six months after discharge: The caregivers were encouraged to persevere *via* IPHE. A questionnaire-based evaluation was conducted on the caregivers, based on which individualized professional health education was provided according to each caregiver’s problems during ostomy care. In addition, a systematic evaluation of patient care was conducted to foster a positive attitude toward life for the sake of better caring for the patients, thus promoting family harmony and patient rehabilitation.

### Evaluation indexes

With respect to psychological endurance, we used the Zarit Caregiver Burden Interview (ZBI) and the Simplified Coping Style Questionnaire (SCSQ) to evaluate the caregivers’ degree of burden and level of pressure coping. The score range for ZBI ranges from 0 to 88; the higher the score, the more severe the burden. In addition, a total score of less than 20 represents no burden, 20–39 no burden to mild burden, 40–59 light to moderate burden, and 60–99 severe burden. The positive coping dimension in the SCSQ scale consists of items 1–12, which mainly reflects the characteristics of individuals adopting positive coping methods when encountering stress. The higher the score, the more positive the coping; It reflects the characteristics of individuals adopting negative coping styles when encountering stress. The higher the score, the more negative the coping. With respect to the nursing abilities, we conducted an interview 6 months after discharge to evaluate urinary ostomy complications, including the incidences of uric acid crystals, stoma recession, stoma mucosa granuloma and dermatitis around the stoma by a specialist on the research team.

### General data questionnaire

Based on a literature review and related studies, a questionnaire was designed to collect general data, including the patients’ general information, diagnosis, classification and medical expenditures, and the caregivers’ general information, including age, sex, religion, marriage, educational background, monthly income, and chronic diseases.

### Zarit Caregiver burden interview

In this study, we used the modified ZBI translated by Wang Lie, and Cronbach’s α was 0.872. This questionnaire consists of 22 items, including personal and social burden dimensions and the impact of the care evaluation on caregivers’ physiological, psychological, social, and emotional states. Each item was composed of 5 dimensions, from “No” to “Always”, on a 0 to 4 scale. A higher total score indicated a heavier care burden.

### Simplified coping style questionnaire

Simplified coping style questionnaire (SCSQ) was used to assess the coping level of the caregivers. It consists of positive and negative coping dimensions, totaling 20 items. Based on a 0∼3 score scale, the positive dimension ranged from 0 to 36 points, and the negative dimension was from 0 to 24. SCSQ has been commonly used in various studies in China, exhibiting good reliability and consistency. The Cronbach’s α value was 0.90.

### Family care satisfaction scale

Family care satisfaction scale (FCSS) is based on the degree of satisfaction with the care work of the caregivers at home, where 91–100 indicates very satisfied, 81–90 generally satisfied, 71–80 satisfied, and 61–70 passed. The satisfaction rate = very much satisfied + generally satisfied. The FSCC Cronbach’s α value was 0.935.

### Data collection methods

Using a standardized instruction language, an investigation was conducted on the caregivers 1 day after the urinary ostomy and at 3 and 6 months after surgery. All the subjects were required to fill out the FCSS, ZBI, and SCSQ forms. When filling out these forms, the investigators would answer questions or give explanations but could not fill out the forms for the caregivers. The completed forms were collected on the scene. A total of 80 questionnaires were distributed, and all were recovered, with a response rate of 100%.

**FIGURE 2:**
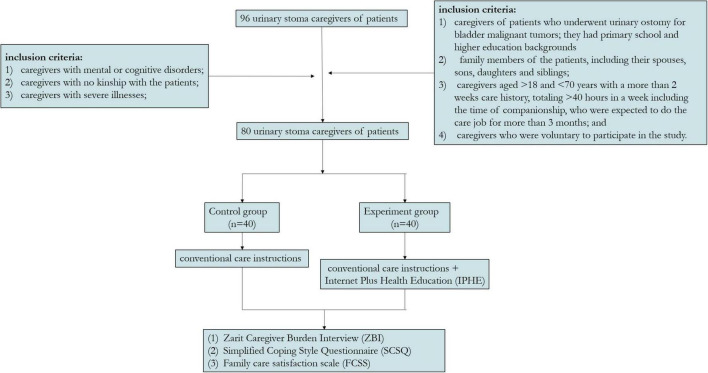
The implementation steps of this study.

Six months after the urinary ostomy, the research team conducted another investigation on the occurrence of ostomy complications by using the standardized instruction language. The intervention steps of this study are shown in Figure 2.

### Statistical analysis

All data were analyzed by SPSS26.0. Measurement data were expressed as x ± s. For normally distributed data, a comparison between two groups was conducted by *t*-test of two independent samples, and a comparison of the same group before and after the intervention was conducted by paired *t*-test. ANOVA was used to test the significance of the difference between the means of the same group at different intervention times (> 2). Counting data were expressed as n (%) and verified by the rank sum test. A *p*-value less than 0.05 was statistically significant.

## Results

### Comparison of general data between the two groups

There was no significant difference in sex, age, body mass index (BMI), educational background and relationship between the two groups at baseline (*P*_s_ > 0.05) (Table 1).

**TABLE 1:** Basic information about investigators.

	Control (*n* = 40)	Experiment (*n* = 40)	χ^2^	*P*
Sex			0.818	0.366
Male	15	19		
Female	25	21		
Average age, year	45.28 ± 5.39	43.87 ± 7.26	0.986	0.327
Average BMI	20.58 ± 2.22	20.12 ± 3.39	0.718	0.475
Educate			–0.164	0.869
Elementary	12	14		
Junior/high	18	15		
University degree	10	11		
Relationship with patients			–0.304	0.761
Spouse	23	25		
Child	13	10		
Other	4	5		

### Internet plus health education effectively reduces the burden on caregivers

**FIGURE 3:**
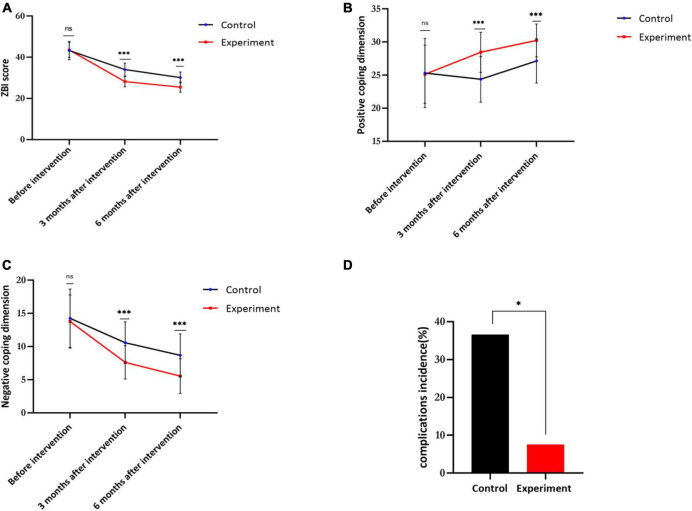
Visualization of the results of this study. **(A)** The Zarit Caregiver burden interview (ZBI) score of control and experiment groups. **(B)** The positive coping dimension of control and experiment group. **(C)** The negative coping dimension of control and experiment groups. **(D)** The complications incidence of control and experiment groups (*n* = 40, *p < 0.05, **p < 0.01; ***p < 0.001; ns, non-significant).

There were significant differences in ZBI scores between the two groups at baseline and 3 and 6 months after the intervention (*P*_s_ < 0.005). The ZBI scores were significantly lower in both groups at 3 and 6 months after the intervention (*P*_s_ < 0.005). Furthermore, the ZBI scores in the experimental group were significantly lower than in the control group at 3 and 6 months after intervention (*P* < 0.005) (Figure 3A and Table 2).

**TABLE 2:** Comparison of Zarit Caregiver burden interview (ZBI) scores between the two groups before and after intervention (x ± s).

Group	Control (*n* = 40)	Experiment (*n* = 40)	*t*	*P*
Before intervention	43.26 ± 4.48	43.55 ± 3.62	0.318	0.751
3 months after the intervention	34.02 ± 3.15^A^	28.19 ± 2.55^A^	9.098	<0.001
6 months after the intervention	30.15 ± 2.67^B^	25.48 ± 2.47^B^	8.120	<0.001
*F*	146.700	443.300		
*P*	< 0.001	<0.001		

^A^Compared with before intervention within the group, compared with 3 months after the intervention within the same group, both *P* < 0.05.

### Comparison of simplified coping style questionnaire scores between groups before and after intervention

There were significant differences in positive and negative dimension scores between the two groups before and after intervention (*P* < 0.005). Further pairwise comparison showed that the positive coping dimension scores at 3 and 6 months after intervention were significantly higher than at baseline in both groups (both *P* < 0.005), while the negative coping scores at 3 and months after intervention were significantly lower (both *P* < 0.005).

The positive coping dimension scores in the experimental group were significantly higher than in the control group at 3 and 6 months after intervention (both *P* < 0.005), while the negative coping dimension scores in the experimental group were significantly lower than in the control group at 3 and 6 months after intervention (both *P* < 0.005) (Figures 3B,C and Table 3).

**TABLE 3:** Comparison of simplified coping style questionnaire (SCSQ) scores between the two groups before and after intervention (x ± s).

Group	Before intervention	3 months after the intervention	6 months after the intervention	*F*	*P*
**Positive coping dimension**					
Control (*n* = 40)	25.31 ± 5.22	24.37 ± 3.46^C^	27.14 ± 3.35^D^	4.720	0.011
Experiment (*n* = 40)	25.13 ± 4.39	28.45 ± 3.02^C^	30.24 ± 2.49^D^	23.320	<0.001
*t*	0.167	5.619	4.697		
*P*	4.697	<0.001	<0.001		
**Negative coping dimension**					
Control (*n* = 40)	14.26 ± 4.41	10.58 ± 3.12^C^	8.67 ± 3.22^D^	24.490	<0.001
Experiment (*n* = 40)	13.78 ± 4.02	7.61 ± 2.51^C^	5.54 ± 2.61^D^	75.330	<0.001
*t*	0.509	4.691	4.776		
*P*	0.612	<0.001	<0.001		

^C^Compare with the same group before intervention in the same dimension, ^D^Compared with the same group in the same dimension for 3 months, all *P* < 0.05.

### Internet plus health education significantly reduces the incidence of urinary stoma complications

As shown in Table 3, the caregivers receiving OPHE demonstrated higher stoma care skills than those receiving conventional training, indicating that urinary stoma care in the experiment group was more professional and meticulous, thus significantly reducing the incidence of ostomy complications (Figure 3D and Table 4).

**TABLE 4:** Comparison of postoperative complications between the two groups (%, x ± s).

Group	Control (*n* = 40)	Experiment (*n* = 40)	*P*
Uric acid crystals	10	2.5	
Stoma recession	7.5	0	
Stoma mucosa granuloma	4	0	
Dermatitis around stoma	15	5	
Overall incidence	36.5	7.5	<0.05

## Discussion

### Internet plus health education reduces the care burden of urinary stoma caregivers

The present study results showed that IPHE could significantly reduce the care burden of urinary stoma caregivers. Given the unique characteristics of urinary ostomy patients, the health education of their caregivers is essential. Furthermore, the degree of acceptance of health education is largely affected by psychological and emotional states. In addition, family economic pressure, social work demand and allocation of time and energy levels challenge the continuity and systematicity of health education (Gönen et al., 2019; Stelton, 2019). The conventional education mode often fails to attract the attraction to caregivers. In addition, busy nursing work in most surgical departments of tertiary hospitals can only spare limited time for health education, and therefore it is usually difficult to find suitable hours to implement health education on both patients and their caregivers continually and systematically (Burkhard et al., 2006). In addition, in the context of the COVID-19 epidemic, many patients find it challenging to go to the hospital (Barbarot and Stalder, 2014). The IPHE mode provides a novel approach to providing adequate post-discharge health education to patients and caregivers. Indeed, caregivers can receive professional health knowledge and comprehensive care instructions without time and location constraints to compensate for their lack of knowledge.

### Internet plus health education raises the positive pressure coping dimension of the urinary stoma caregivers

Most caregivers in this study were middle-aged adults about 45 years old with a middle school educational background. With the popularity of the internet and the wide application of smartphones, they can use the WeChat platform by allocating their time appropriately for fragmental learning to obtain necessary knowledge and information in urinary stoma care (Cook et al., 2008; Tregoning et al., 2021). Current evidence suggests (Calvillo-Arbizu et al., 2021) that a positive coping mode reduces the care burden of the urinary stoma caregivers, while a negative coping mode increases their care burden. Importantly, the IPHE mode combines education with entertainment by using presentations, comics, videos and interactive games based on an abundant network and multi-media resources (Cohen et al., 2021), which is beneficial to both the patients and caregivers to heighten their knowledge and skills about stoma care and compensate for the deficits of conventional supply-oriented didactic health education, thus making health education more standardized and colorful. Internet resources are reproducible and can be used without time constraints (Chen et al., 2022; Coughlin et al., 2022). It can provide more extensive communication, reduce time consumption, improve the quality of care, and contribute to establishing a “Healthy China” in the Internet environment. The researchers of the present study progressively conducted health education to assist caregivers in correctly assessing the condition of the stoma and the patients’ behavior in different stages, teach them stoma care skills and instruct them how to communicate with the patients properly to reduce the psychological burden during the long-term care process, help them accept their role as a caregiver to increase their sense of self-efficacy and improve their care skills.

### Internet plus health education effectively enhances the stoma care ability of the caregivers

The present substantiated that IPHE could enhance the caregivers’ stoma care ability. The IPHE described herein harnesses language, audio and video of modern multi-media communications technologies to help the caregivers in all aspects and learn about their main and difficult points in postoperative urinary stoma care (Henson et al., 2022). In addition, this IPHE mode is appropriate to our current pace of life and work and helps the caregivers learn and master the main nursing points through fragmented time to reduce the tiredness of the caregivers during the learning process (Baumeister et al., 2022; Eke et al., 2022). More importantly, it can effectively reduce the occurrence of postoperative complications. Postoperative complications are common phenomena after urinary ostomy and are the main and difficult points in postoperative care (Heerschap and Duff, 2021; Hobbs et al., 2022). As described in the previous section, the IPHE mode reduced uric acid crystals, stoma recession, stoma mucosa granuloma and dermatitis around the stoma, thus greatly improving patient quality of life, reducing the re-hospitalization rate, and ensuring optimal use of medical resources.

Our study showed that IPHE mode reduced the care burden of urinary soma caregivers effectively, raised the positive pressure coping dimension and significantly improve caregivers’ nursing ability. The care of urostomy required professional medical knowledge and nursing skills, but this was difficult to achieve for the patients’ caregivers. Therefore, the necessity of our study was illustrated. Secondly, Our research reflected the contemporary value. In the age of COVID-19, many of the restrictions on hospital access exist for patients and their caregivers. Compared with face-to-face health education, the application of IPHE mode greatly facilitates patients and their caregivers, which makes the communication between patients and doctors not limited to time and place. In addition, our research has robust utility. Based on the working model of IPHE, caregivers establish a closer connection with the hospital, which also helps them reduce the burden of caring for patients and increase their enthusiasm for care. The repeated health education of caregivers in the IPHE model has significantly improved the care level of caregivers and reduced the incidence of patients’ complications. Finally, this model was not limited to urostomy patients, but extends to patients with other diseases, showing the applicability of our study.

However, there were some limitations in our study. First, this was a single-center study with a relatively small study population size which limited the generalizability of our findings. Moreover, evaluation in our research was based on scale measures, and the objectivity of evaluation was insufficient, warranting further studies. Finally, whether Internet Plus Health Education can be used on a large scale and its long-term results still need to be assessed in future studies.

## Conclusion

The IPHE mode described in this study improved the post-discharge care ability of the urinary stoma caregivers, raised their confidence in caring for the patients, dredged their negative emotions during the caring process, reduced their care burden and pressure with a positive attitude, minimized the occurrence of urinary ostomy complications, and improved patient quality of life. Overall, extensive use of the IPHE mode can assist the implementation of the “Healthy China” plan and help urinary ostomy patients and their caregivers.

## Data availability statement

The raw data supporting the conclusions of this article will be made available by the authors, without undue reservation.

## Ethics statement

The studies involving human participants were reviewed and approved by Ethics Committee of The First Affiliated Hospital of Wenzhou Medical University. The patients/participants provided their written informed consent to participate in this study.

## Author contributions

XF, HX, and HL conceived and designed the study. HL, LL, XZ, and HX performed the literature search. HX and XF for experimental design and implementation. HX, LL, and HL analyzed the data. XF and HL wrote the manuscript and contributed equally to this work. HX reviewed the manuscript and supervised the research. All authors have read and approved the manuscript.
